# Optimizing daylily (*Hemerocallis citrina* Baroni) cultivation: integrating physiological modeling and planting patterns for enhanced yield and resource efficiency

**DOI:** 10.3389/fpls.2024.1442485

**Published:** 2024-09-13

**Authors:** Weijia Li, Kun Zhang, Jianxia Liu, Juan Wu, Yue Zhang, Michael Henke

**Affiliations:** ^1^ Engineering Research Center of Coal-Based Ecological Carbon Sequestration Technology of the Ministry of Education, Shanxi Datong University, Datong, China; ^2^ Key Laboratory of Graphene Forestry Application of National Forest and Grass Administration, Shanxi Datong University, Datong, China; ^3^ College of Agronomy and Life Science, Shanxi Datong University, Datong, China; ^4^ College of Agronomy, Hunan Agricultural University, Changsha, China

**Keywords:** daylily (*Hemerocallis citrina* Baroni), functional-structural plant model (FSPM), planting patterns, crop growth simulation, source-sink relationship, yield simulation, land use efficiency

## Abstract

**Introduction:**

Optimizing the dynamics of daylily (Hemerocallis citrina Baroni) growth under various planting patterns is critical for enhancing production efficiency. This study presents a comprehensive model to simulate daylily growth and optimize planting patterns to maximize bud yield while minimizing land resource utilization.

**Methods:**

The model incorporates source-sink relationship specific to daylilies into physiological process modeling, considering environmental factors such as micro-light and temperature climate, and CO2 concentration. Spatial factors, including planting pattern, row spacing, plant spacing, and plant density were examined for their impact on light interception, photosynthesis, and resource efficiency. Employing partial least square path modeling (PLS-PM), we analyzed the interrelations and causal relationships between planting configurations and physiological traits of daylily canopy leaves and buds. Through in situ simulations of 36 planting scenarios, we identified an optimal configuration (Scenario ID5) with a density of 83,000 plants·ha^−1^, row spacing of 0.8 m, and equidistant planting with a plant spacing of 0.15 m.

**Results and discussion:**

Our research findings indicate that increased Wide+Narrow row spacing can enhance yield to a certain extent. Although planting patterns influence daylily yield, their overall impact is relatively minor, and there is no clear pattern regarding the impact of plant spacing on individual plant yield. This modeling approach provides valuable insights into daylily plant growth dynamics and planting patterns optimization, offering practical guidance for both farmers and policymakers to enhance daylily productivity while minimizing land use.

## 1 Introduction

Daylily (*Hemerocallis citrina* Baroni), also known as the golden needle vegetable or forget-one’s-sadness plant, is a perennial herbaceous plant of the *lily* family, cultivated throughout northern and southern China (Lim, 2015). The edible daylily buds are rich in proteins, carbohydrates, fats, vitamins, and various amino acids. They are highly valuable as nutritionally rich food and also have notable medicinal uses for treating various diseases (Li et al., 2017; Tian et al., 2017). In Datong, Shanxi Province in the north of China, a significant region for daylily production, local farmers refer to these flowers as the “wealth flower” due to their economic importance. As a specialty vegetable with substantial economic benefits, increasing the yield of daylilies is crucial. Despite the economic significance of daylilies, there has been a lack of advanced research focused on optimizing their growth through precise simulation models.

Different planting configurations directly influence the light micro-climate within the plant canopy, leading to interplant shading. Suboptimal configurations hinder the canopy leaves’ access to optimal light supply and local micro-temperature climate conditions, both of which are directly related to photosynthesis and growth (Slattery and Ort, 2021). Suboptimal conditions lead to unnecessary light competition between plants, typically resulting in increased vegetative growth at the expense of flower and fruit yield (He et al., 2020). In rice cultivation, it has been demonstrated that increasing the distance between individual seedlings can improve yield under certain water conditions (Mishra and Salokhe, 2010). A decrease in yield can also occur gradually with increasing density in corn cultivation (Murphy et al., 1996). For daylily plant architecture, the light distribution within the canopy primarily depends on internal plant characteristics such as the number of tillers, plant height, plant width, number of main stem leaves, leaf length, leaf width (widest part diameter), scape length (length from the base of each scape to the lower end of the inflorescence), number of buds (total number of buds on each scape during the entire flowering period), and bud length, all of which are highly spatiotemporally variable. Changes in planting configuration and leaf growth can alter the spatial position of the leaves, leading to changes in light interception and photosynthetic rate, which in turn affect the carbon assimilation of daylily plants and ultimately the yield of daylilies (buds). Despite these complexities, existing models lack precision in simulating canopy changes related to plant configuration, often failing to accurately capture dynamic light and temperature interactions (Rötter et al., 2015).

Functional–structural plant modeling (FSPM) provides a well-established approach to simulate three-dimensional growth models, improving our understanding of morphological, physiological, and biological processes driving crop development, growth, and yield formation (Vos et al., 2010). It also simulates the interaction between crops and their environment under various conditions, including the effects of biotic and abiotic stresses (Soualiou et al., 2021). The primary advantage of FSPM lies in its detailed simulation of plant morphogenesis, three-dimensional structure, and architectural development, leading to higher accuracy of simulation and prediction. Many current FSP models employ static models for simulation, where key physiological processes are derived from traditional crop models. This approach neglects the dynamic development of plant growth and changing architecture. As a result, although these static FSP models can somewhat simulate the complex light distribution within the canopy structure, they struggle to accurately capture dynamic growth behaviors, leading to less reliable yield predictions (Zhu et al., 2015; Zhang et al., 2020a).

In addition to morphogenesis and plant morphology modeling, FSPM is widely used to integrate plant physiological processes on spatial and temporal scales. Notably, it has been used to explore the mechanism of assimilating carbohydrates through photosynthesis (Zhu et al., 2013) and to address the dynamic allocation of photosynthetic products between plant organs (Sonnewald and Fernie, 2018; Lacointe and Minchin, 2019). The simulated effects of CO_2_ fertilization and changes in canopy structure on soybean’s gross primary productivity (GPP) have been studied (Song et al., 2020). Regarding the dynamic simulation of the allocation of photosynthetic products, a three-dimensional growth model of rice was established by integrating source–sink relationships and quantitative trait locus (QTL) information (Xu et al., 2009). Carbon allocation models applicable to static tree structures, namely, the multi-scale carbon allocation model (MuSCA) and the autonomous units carbon allocation model (AUCAM), have also been developed (Auzmendi and Hanan, 2020; Reyes et al., 2020). The allocation of carbon from source to sink organs is driven by sink strength, and studies of poplar and potato varieties have highlighted the significance of source–sink coordination in determining biomass productivity (Balasubramanian et al., 2023; Liu et al., 2023). As such, FSPM models based on source–sink relationships have been proven to accurately and exhaustively simulate the dynamic growth of plants in other crops. However, the application of FSPM methods to daylilies has not yet been seen.

In summary, current research indicates that there has been no explicit involvement in simulation research related to daylilies so far; also, combining the robust micro-environment simulation capabilities of FSPM with the powerful carbon allocation calculations of source–sink relationship models can greatly enhance simulation accuracy, thereby allowing for a more precise analysis of the effects of different planting patterns on daylily plants. Daylilies, as a species of *Hemerocallis*, have unique biological characteristics, including strong adaptability to light and drought tolerance (Xie et al., 2022) and a lower photosynthetic rate compared to field crops such as wheat and rice (Geng et al., 2023), which are significantly different from other economic crops. Therefore, it is crucial to investigate the physiological growth processes and bud yield of different planting patterns for daylilies to enhance land-use efficiency. This study aims to i) investigate the effects of different planting configurations on solar radiation interception and photosynthesis at the plant and organ levels in daylilies, ii) examine how changes in planting configuration affect the bud yields of daylilies under various planting densities, and iii) propose an optimal planting pattern that enhances solar radiation interception and photosynthesis while maximizing bud productivity in the context of plant competition.

## 2 Materials and methods

### 2.1 Data collection and processing

The field experiment was conducted from March to August 2022 in the Tangjiapu Organic Daylily Standardized Planting Base, Yunzhou District, Datong, Shanxi Province. The base is located at coordinates 40°08′N, 113°54′E, characterized by a temperate continental semiarid monsoon climate with an average annual temperature of 6.4°C and annual precipitation of 439.9 mm. The soil type at the site is volcanic soil, known for its high zinc and selenium content. The tested variety, “Datong” variety (*Hemerocallis citrina* Baroni), is a popular and widely utilized cultivar known for its distinctive yellow flowers. This variety is favored for its high yield and local adaptability, making it a valuable choice for agricultural production. Its reliability and productivity have established it as a commonly used selection among growers. The experiment, conducted on a total area of 4 ha, employed initial row spacing of 1.2 m and plant spacing of 0.2 m, resulting in a planting density of 72,000 plants per hectare. The tested, yellow-flowered daylilies were 3 years old with approximately six shoots per clump.

To ensure the practical applicability and effectiveness of the optimized planting patterns for enhancing daylily production and resource efficiency, the simulation results were validated through field trials. Eight representative daylily plants were randomly selected in the experimental field for individual plant measurements. Morphological data of the plants were collected every 3 days throughout the entire growing season of 5 months, leading to 48 measurements. The daily average solar radiation intensity was 248.7 W·m^−2^, with an average sunshine duration of 5.9 h, an average temperature of 19.1°C, an average cloud cover of 0.4, and an average wind speed of 2.4 m/s. Based on the growth characteristics of the plants, the entire growth period was divided into three stages: spring seedling growth stage (early April to mid-May), scape emergence and bud stage (late May to mid-June), and flowering and harvesting stage (late June to mid-August). Leaf markers were applied to each leaf using instruments such as vernier calipers, tape measures, and analytical balances to measure shoot number, plant height, plant width, number of main stem leaves, leaf length, leaf width (maximum transverse diameter at the widest part), flower scape length (from the base of each flower scape to the lower end of the inflorescence), number of buds (total number of buds on each flower scape throughout the entire flowering period), bud length, and fresh and dry weight of the buds. The emergence points of the leaves, scapes, and buds at different leaf positions of the daylilies were recorded, and the growth status of the abovementioned organs was monitored every 3 days. Additionally, field-measured biomass data are collected concurrently with morphological measurements to obtain comprehensive growth data. By comparing the simulated outcomes with the actual field data, we can verify the model’s reliability and make necessary adjustments to optimize planting patterns further.

### 2.2 Virtual model construction

#### 2.2.1 Modeling environment

The presented functional–structural plant model (FSPM) was implemented using the interactive modeling platform GroIMP (Kniemeyer, 2008), an open-source software freely available from GitLab (https://gitlab.com/grogra/groimp). GroIMP integrates a Java-based modeling language called extended L-system language or XL for short (Kniemeyer, 2008), specially designed to support all needs of functional–structural plant modeling (Vos et al., 2007), including full-spectral light modeling (Henke and Buck-Sorlin, 2017), or an integrated solver for ordinary differential equation (Hemmerling, 2012; Hemmerling et al., 2013).

#### 2.2.2 FSP model description

**Figure 1:**
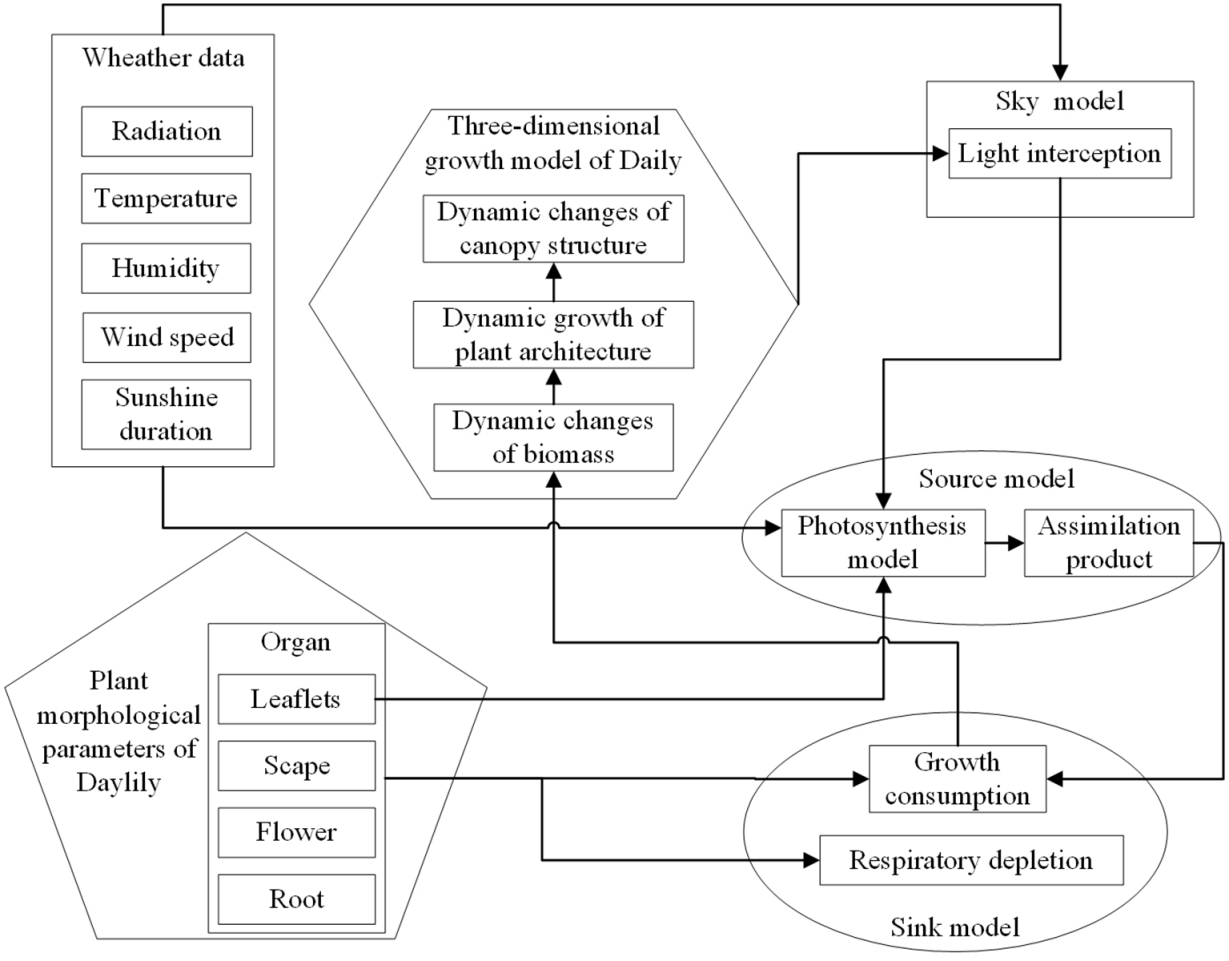
Schematic diagram of the modular setup, hierarchical structure, and main inputs and outputs of the daylily FSPM.

The constructed FSPM consists of various dedicated submodules including 1) the overall control model (e.g., plant and scene initiation, summarize and output modules), 2) the direct and diffuse sun and sky radiation model, 3) the morphology model, 4) the photosynthesis model, and 5) the source–sink model. In this model, all other submodules are integrated as external extension models within the main overall control model. Based on field experimental data, this study established quantitative relationships among leaf growth morphology, photosynthesis, source–sink relationships, and yield, thus constructing a simulation model for growth. **Figure 1** provides an overview of the FSPM model structure and the relations between the included model components (modules).

Using meteorological data as model input, leaf photosynthesis serves as the main source term and assimilates products as increments to the sink model for organ growth consumption. As virtual plants grow dynamically, changes in leaf light interception lead to dynamic adjustments in leaf temperature, thereby influencing the rate at which leaves fix environmental CO_2_, i.e., affecting photosynthetic rates within the canopy. These changes further impact variations in source–sink relationships within the biomass of plant organs. During model initialization, global parameters and variables are loaded, and the sky radiation model along with the initial parameters of plant populations is imported into the scene.

The specific iteration process involves a main loop, successively executing individual growth steps until the intended growth period of day 90 to 242 is simulated. The temporal resolution of the simulation is set to 1 day. During each simulation step, five subprocesses are executed: updating the sky radiation model, calculating the single leaf photosynthesis module, running the source–sink model, applying rules, and updating the output morphology simulation model. Finally, statistical outputs such as organ biomass are generated.

#### 2.2.3 Sun and sky radiation module

The implemented sun and sky radiation model utilized in this study follows the description given in Buck-Sorlin et al. (2011), specifically adopting their approach for the arrangement of light sources, the simulation of diffuse sky radiation, and the configuration of the direct sunlight parameters. It consists of 72 direct light sources, arranged in a hemisphere, to simulate a more realistic light environment by representing the diffuse sky (which represents scattered light from the atmosphere), and one direct light source simulating the direct sunlight (the unscattered solar rays that directly reach the Earth’s surface). The integrated ray tracer is based on a reverse Monte Carlo path tracer (Hemmerling et al., 2008). This model facilitates the computation of the intercepted light radiation within the scene by simulating both direct radiation sources (such as the sun) and diffuse, scattered radiation sources (such as the sky). For realistic and reproducible results, 200 million rays are simulated during each (light) simulation step. This specific number was chosen based on sensitivity tests, which showed that using approximately 200 million rays provides a good balance between computational time and the quality of light distribution. The detailed modeling approach follows the methodology outlined by Zhang et al. (2020b). To more accurately simulate light radiation under cloudy conditions, in addition to the clear sky scenario described above, a cloud cover-based solar radiation model (CSRM) is incorporated to simulate daily average solar radiation under overcast weather conditions (Ahamed et al., 2022), as shown in Equation 1.


(1) $$\frac{H}{H_{\text{o}}}=-0.08+0.21{\left(T_{\text{max}}-T_{\text{min}}\right)}^{0.5}-0.012\overset{ˇ}{N}$$

Where $T_{\text{max}}$ represents the daily maximum temperature in degrees Celsius, $T_{\text{min}}$ represents the daily minimum temperature in degrees Celsius, $H_{\text{o}}\;$ represents the daily average extraterrestrial radiation in W·m^−2^, and $\overset{ˇ}{N}$ denotes the average cloud cover observed by the local meteorological station during the day.

The validation data for the sky radiation model are verified using hourly meteorological data from the Yuzhou District Meteorological Station in Datong City, Shanxi Province (Yamaltu Big Data Information (Ningbo) Co., Ltd.). The model’s evaluation metrics include a correlation coefficient (*r*) of 0.93, an *R*² of 0.87, and a root mean squared error (RMSE) of 28.52 W/m², which accounts for approximately 9.3% of the measured total radiation. These results indicate a strong agreement between the model’s output and the observed physical measurements (**Supplementary Figure S1**). For a detailed verification process, please refer to Zhang et al. (2024).

#### 2.2.4 The construction of daylily morphology simulation module

**Figure 2:**
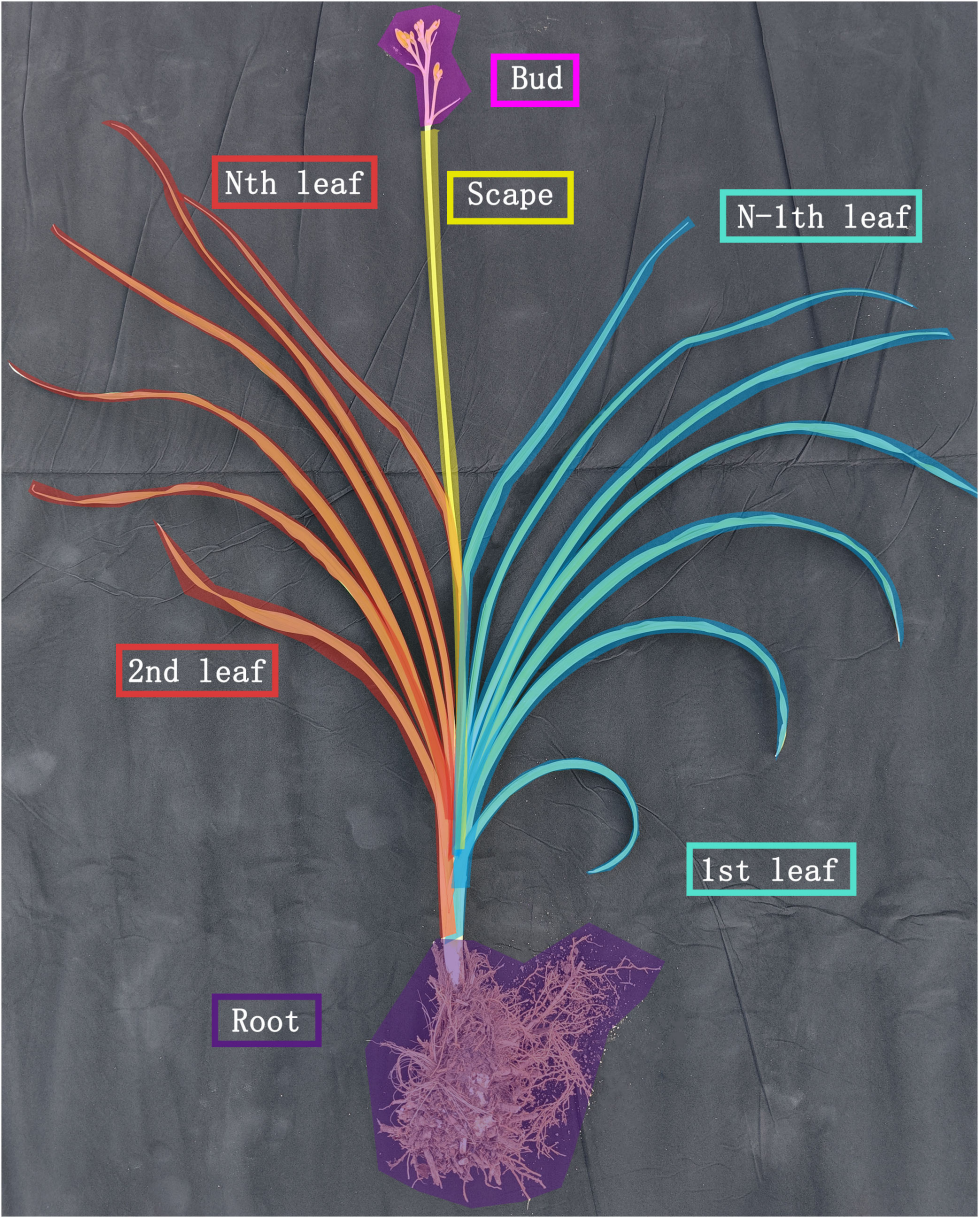
Schematic of the definition of daylily phytomers. Daylily phytomers including the opposite leaves on the plant (red and blue boxes), scape (yellow box), and flower bud attached to the scape (pink box).

The actual morphological plant model of a single daylily plant consists of defined plant organs, i.e., internodes, leaves, flower stem (scape), and bud organs. Leaves are constructed as modules containing attributes, e.g., to store the intercepted radiation, current dry weight, and organ age. They are visualized in the 3D scene as a sequence of parallelograms of varying directions and sizes following the measured data of the real-world experiment. Upon the formation of each leaf, it is assigned a rank label, which is used to track its developmental stage and position on the plant (**Figure 2**), and its absorbed radiation amount (W·m^−2^) is determined using the sun and sky radiation model, subsequently converted into photosynthetic photon flux density (μmol PPFD·m^−2^·s^−1^) with a conversion factor of 2.275. This factor is chosen for its accuracy in reflecting the relationship between radiative energy and photosynthetically active radiation, consistent with values reported in similar studies (Henke et al., 2016).

The growth and expansion rates of each organ of the plant are described using the β growth function (Yin et al., 2003). This function delineates the dynamics of organ expansion and biomass accumulation: as simulation time progresses, the function is applied to all virtual organs, thereby facilitating the overall growth of the virtual plant. For instance, the size of the leaves is simulated using the β growth function, with biomass increment calculated through leaf photosynthesis serving as the final leaf length and growth time. Subsequently, the increment in leaf area is computed using the specific leaf area (SLA) as a constant (SLA = 0.0023 m² leaf·g^−^¹ leaf), which was calculated from the field measurements. The use of the SLA method for this calculation is based on the approach described by Yin and van Laar (2005), as shown in Equation 2.


(2) area = SLA × biomass

In this context, “area” refers to leaf area, while “biomass” represents the increase in leaf dry matter. In summary, given the input climate data, the model can simulate the dynamic changes in the canopy or individual plant morphological growth and biomass (dry matter) accumulation, as well as the final yield of daylily buds.

#### 2.2.5 Source–sink module

As the sink organs grow and develop, they drive the conversion of assimilates into harvestable dry matter, namely, the yield of daylily buds. The FSPM source–sink relationship model in this study is established based on the rice relationship model developed by Xu et al. (2009), where the sink activity rules are determined by the growth and development rules of each organ (β growth function) (Yin et al., 2003), and the real-time potential sink strength $S_{pot}^{str}$ can be expressed by Equation 3.


(3) $$S_{pot}^{str}=\frac{dw}{dt}=c_{m}\left(\frac{t_{e}-t}{t_{e}-t_{m}}\right){\left(\frac{t}{t_{m}}\right)}^{\frac{t_{m}}{t_{e}-t_{m}}}$$

Here, $c_{m}$ represents the maximum growth rate during the linear growth phase at time point $t=t_{m}$ , where c represents the growth rate, and *t* is the time variable. The variable $t_{e}$ denotes the moment when organ growth ceases (i.e., growth rate becomes zero) upon reaching maximum size or mass (*w*). The overall carbon demand ( $S_{tot}^{dem}$ ) is the weighted sum over time of the potential growth rates of all virtual organ structures according to the source–sink relationship rules, as illustrated in Equation 4.


(4) $$S_{tot}^{dem}=\sum \;S_{pot}^{str}\Delta t$$

The actual growth rate, denoted as $gr_{real}$ , is obtained by multiplying the current shared assimilate pool size, denoted as *ap*, by the potential-real growth rate $\;S_{real}^{str}$ . This ensures that $gr_{real}$ does not exceed $S_{pot}^{str}$ , as depicted in Equation 5.


(5) $$ɡr_{real}=S_{real}^{str}ap=\frac{S_{pot}^{str}}{S_{tot}^{dem}}ap\left(ɡr_{real}\leq S_{pot}^{str}\right)$$

Once organs grow at their actual growth rates, the central carbon pool is correspondingly replenished. Lastly, growth respiration is considered in terms of a conversion factor (g glucose·g^−1^ dry matter), which is directly proportional to the growth rate. This factor, derived from Goudriaan and van Laar (1994), reflects the energy cost associated with biosynthesis. Similarly, maintenance respiration is also calculated based on a fixed proportion of structural biomass (0.014 g glucose·g^−1^ dry matter) and accounts for the energy required to sustain existing tissue, based on the same reference (Goudriaan and van Laar, 1994). These specific rates were chosen due to their established accuracy in representing the metabolic costs of growth and maintenance in plant models, and both terms were subtracted from the central pool at each simulation step. For ease of calculation, root assimilate consumption is then computed at a fixed proportion (0.15 × central carbon pool). The computation for each time step subtracts the above items from the central carbon pool. The fresh weight of each organ is calculated by multiplying the dry weight by different empirically determined constants.

#### 2.2.6 Photosynthesis module

This study implemented computational modeling of assimilates in leaf sources using an extended Kim and Lieth module (Kim and Lieth, 2003), which is a general module used to estimate short-term steady-state CO_2_, water vapor, and heat fluxes in C3 plant leaves, explicitly coupling the major physiological processes of photosynthesis [Farquhar, von Caemmerer, and Berry (FvCB) model (Farquhar et al., 1980)], including biochemical assimilation processes, stomatal conductance [Ball, Woodrow, and Berry (BWB) model (Ball et al., 1987)], and leaf energy balance. Specific parameters for daylily species were extracted from literature data to parameterize the photosynthesis model (Geng et al., 2023). Initially, the photosynthesis model was calibrated independently; for model parameter values, please refer to **Supplementary Tables S1**, **S2** for details.

The validation experiment of the daylily photosynthesis model utilized the CIRAS-3 photosynthesis instrument (CIRAS-3, PP Systems, USA) to measure the daylily leaf blades. The instrument was equipped with a 1.75-cm^2^ general-purpose leaf chamber featuring red, green, blue, and white light-emitting diodes (LEDs) (PLC3) and an external CO_2_ cylinder to simulate the light, temperature, air, and humidity conditions required by the leaf blades. Measurements were conducted between 8:30 a.m. and 11:30 a.m. The detailed measurement protocol and verification were the same as in Zhang et al. (2024).

### 2.3 The experimental setup for virtual simulation

**Figure 3:**
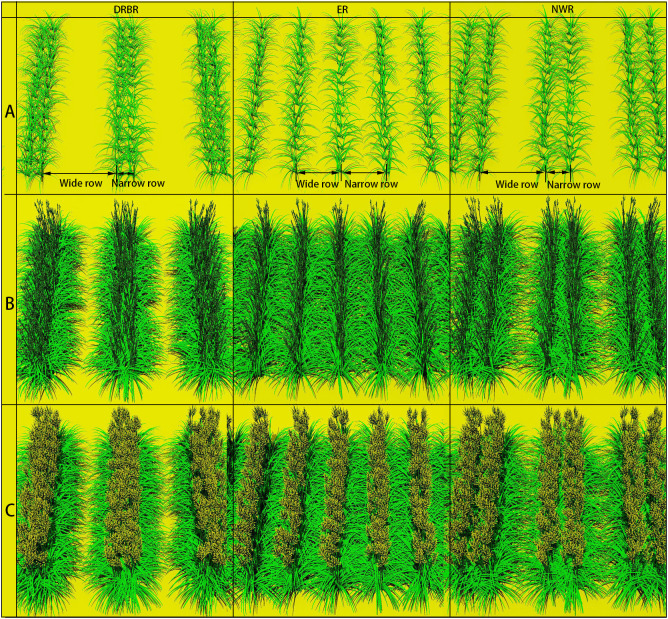
Screenshots of three simulated planting patterns for daylily plants during three growth stages [the seedling growth period **(A)**, the flower scape emergence period **(B)**, and the flowering period **(C)**], namely, the double row big ridge (DRBR), equidistant row (ER), and narrow–wide row (NWR). Corresponding to scenario ID = 13, with a planting density of 72,000 plants·ha^−1^, a wide row spacing of 1.2 m, a narrow row spacing of 0.2 m, and a plant spacing of 0.2 m.

This study simulated a total of 36 most commonly used planting configurations (**Table 1**) under three planting patterns [double row big ridge (DRBR), equidistant row (ER), narrow–wide row (NWR)], six wide row + narrow row patterns (W+N pattern, 0.8–1.8 m), four plant spacings (0.15–0.3 m), and 10 initial planting densities (63,000–100,000 plants·ha^−1^) for daylilies in their third year of growth, whereas the third year is the critical yield formation period, with an average of six shoots per plant, and the flowering scape emergence rate of daylilies was set at 50%. The selection of these planting configurations was based on principles aimed at increasing land-use efficiency, facilitating mechanized planting, and adhering to modular design standards. **Figure 3** illustrates the most common planting density configurations used in local production (72,000 plants·ha^−1^) for DRBR rows, ER rows, and NWR rows, corresponding to scenario IDs 13, 14, and 15 in **Table 1**, respectively. The simulation period was set from 1 April 2022 (day 91 of the year) to 30 August 2022 (day 242 of the year), with a time step of 1 day. The external climate conditions for all scenarios, including solar radiation, temperature, humidity, and CO_2_ concentration, were simulated using measured data input into the sun and sky radiation module created beforehand. This module employs simulated light rays to trace the transmission, reflection, and refraction of each ray encountering daylily plants, calculating and recording the light interception of each leaf in the canopy. Subsequently, other modules were invoked to further calculate leaf temperature, photosynthetic rate (photosynthesis module), real-time source–sink relationships (source–sink relationship module), and the growth status and fresh weight of the leaves, scapes, and buds (daylily morphology simulation module).

**Table 1:** Detailed configurations of the daylily canopy modeling scenarios.

ID	Planting pattern	Wide row (m)	Narrow row (m)	Wide row + narrow row (m)	Plant spacing (m)	Initial planting density (plants·ha^−1^)
1	DRBR	1.6	0.2	1.8	0.15	74,000
2	ER	0.9	0.9			
3	NWR	1.2	0.6			
4	DRBR	1.4	0.2	1.6	0.15	83,000
5	ER	0.8	0.8			
6	NWR	1.1	0.5			
7	DRBR	1.2	0.2	1.4	0.15	95,000
8	ER	0.7	0.7			
9	NWR	1	0.4			
10	DRBR	1.4	0.2	1.6	0.2	63,000
11	ER	0.8	0.8			
12	NWR	1.1	0.5			
13	DRBR	1.2	0.2	1.4	0.2	72,000
14	ER	0.7	0.7			
15	NWR	1	0.4			
16	DRBR	1	0.2	1.2	0.2	83,000
17	ER	0.6	0.6			
18	NWR	0.8	0.4			
19	DRBR	0.8	0.2	1	0.2	100,000
20	ER	0.5	0.5			
21	NWR	0.6	0.4			
22	DRBR	1	0.2	1.2	0.25	67,000
23	ER	0.6	0.6			
24	NWR	0.8	0.4			
25	DRBR	0.8	0.2	1	0.25	80,000
26	ER	0.5	0.5			
27	NWR	0.7	0.3			
28	DRBR	0.6	0.2	0.8	0.25	100,000
29	ER	0.4	0.4			
30	NWR	0.5	0.3			
31	DRBR	0.8	0.2	1	0.3	67,000
32	ER	0.5	0.5			
33	NWR	0.7	0.3			
34	DRBR	0.6	0.2	0.8	0.3	83,000
35	ER	0.4	0.4			
36	NWR	0.5	0.3			

DRBR, double row with big ridge; ER, equidistant row; NWR, narrow–wide row.

### 2.4 Statistical analysis of simulated data

This study used the partial least squares path modeling (PLS-PM) approach to analyze simulated datasets (all datasets were compiled into one single CSV file). PLS-PM is a variance-based structural equation modeling technique that allows for the analysis of complex cause–effect relationships in systems with multiple dependent and independent variables. This method is particularly suited for exploratory research and for building predictive models when theoretical knowledge is complicated. PLS-PM consists of two models: the measurement model, which defines the relationships between simulated data (such as W+N row distance, density, plant distance) and latent variables (e.g., row distance, leaf radiation, or leaf photosynthesis), and the structural model, which specifies the relationships between latent variables. In the realm of agricultural research, PLS-PM has been employed to analyze and predict the impacts of different agronomic practices on crop performance. Studies have utilized PLS-PM to investigate the effects of irrigation techniques on crop yield, the influence of soil amendments on plant growth, and the correlations between canopy structure and photosynthetic efficiency (Xiao et al., 2022; de Morais et al., 2023). By utilizing PLS-PM, this study aims to provide a detailed understanding of how different planting configurations influence the physiological traits of daylily plants, ultimately aiding in the optimization of cultivation practices.

## 3 Results

### 3.1 Simulation and validation of the growth status of various organs of daylily plants at different growth stages and bud yield

**Figure 3** presents the visual representation of plant populations at three growth stages in the daylily FSPM. The model calculates the light interception of each leaf, subsequently utilizing the assimilates produced by leaf photosynthesis as source terms in the storage organ model to supply the growth requirements of various organs such as the leaves, scapes, buds, and roots.

**Figure 4:**
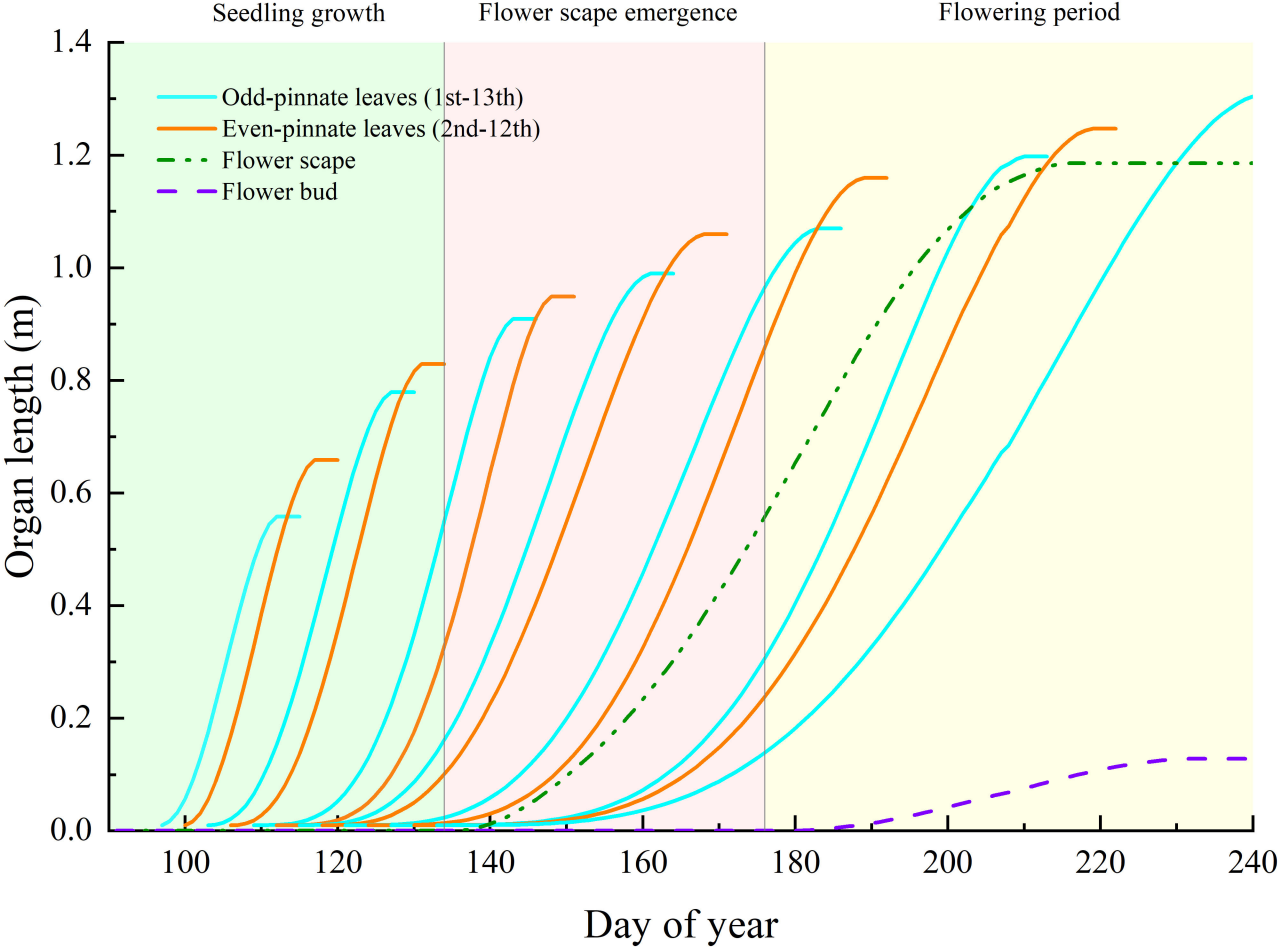
The growth status of each organ throughout the entire plant growth cycle simulated by the daylily FSPM. Note: For better readability of the chart, the length curves are not further drawn shortly after the maximum length is reached.

This study monitored the growth of each daylily leaf, scape, and bud in the order of appearance, calculating the *t_m_* (time of maximum linear growth rate) and *t_e_* (time of growth cessation when the organ reaches its maximum size or mass) using the β growth function of each leaf, scape, and bud. Subsequently, the simulations were conducted based on the final length of the leaves. The monitoring data of the aboveground organs’ growth status in daylily plants were used to calibrate the source–sink relationship model. Since the daylily leaves are narrow and arranged in opposite, with leaf organs appearing in sequence, the time interval between leaf groups is longer than within the opposite pair, resulting in the appearance of paired growth states as shown in **Figure 4**. The simulation of inflorescence growth is similar to that of daylily leaves. The inflorescence begins elongating at the early budding stage (day 137) and grows to its maximum length (1.19 m) by the mid-flowering harvest stage (day 215). The model simulated an average number of 40 buds on the inflorescence, with the average length of the flower increasing from the early flowering harvest stage to the mid-late harvest stage (average length of the buds: 0.128 m, total fresh weight per plant: 171.42 g).

The results, as shown in **Table 2**, indicate that the coefficients of determination (*R* ^2^) between the measured and simulated morphological data of daylily organs range from 0.896 to 0.984, with RMSE values ranging from 0.01 to 0.18 m. The *R* ^2^ for the average fresh weight of buds is 0.88, with an RMSE of 0.50 g. Additionally, the overall sample *F*-values range from 82.24 to 1,168.53, all with significance values less than the significance level of 0.05, indicating a satisfactory degree of model fit.

**Table 2:** Comparison between measured and simulated values of morphological and yield indices of growth processes for various organs in daylily.

Category	*R* ^2^	RMSE	*F*-value	Significance
Leaf rank1	0.98	0.03	304.05	1.14 × 10^−5^
Leaf rank2	0.94	0.06	82.24	2.73 × 10^−4^
Leaf rank3	0.98	0.06	344.19	3.28 × 10^−7^
Leaf rank4	0.94	0.08	106.81	1.72 × 10^−5^
Leaf rank5	0.92	0.14	109.31	1.06 × 10^−6^
Leaf rank6	0.94	0.11	166.14	5.56 × 10^−8^
Leaf rank7	0.92	0.18	158.66	5.01 × 10^−9^
Leaf rank8	0.90	0.14	138.52	2.72 × 10^−9^
Leaf rank9	0.92	0.15	209.28	1.04 × 10^−11^
Leaf rank10	0.92	0.14	241.89	1.23 × 10^−12^
Leaf rank11	0.95	0.15	453.90	5.51 × 10^−18^
Leaf rank12	0.90	0.16	261.31	9.91 × 10^−16^
Leaf rank13	0.92	0.14	406.90	1.70 × 10^−20^
Flower scape length	0.97	0.09	1,168.53	6.49 × 10^−28^
Average bud length	0.91	0.01	212.88	4.01 × 10^−12^
Average bud weight	0.88	0.50	147.55	1.10 × 10^−10^

### 3.2 Daylily canopy configuration analysis

**Figure 5:**
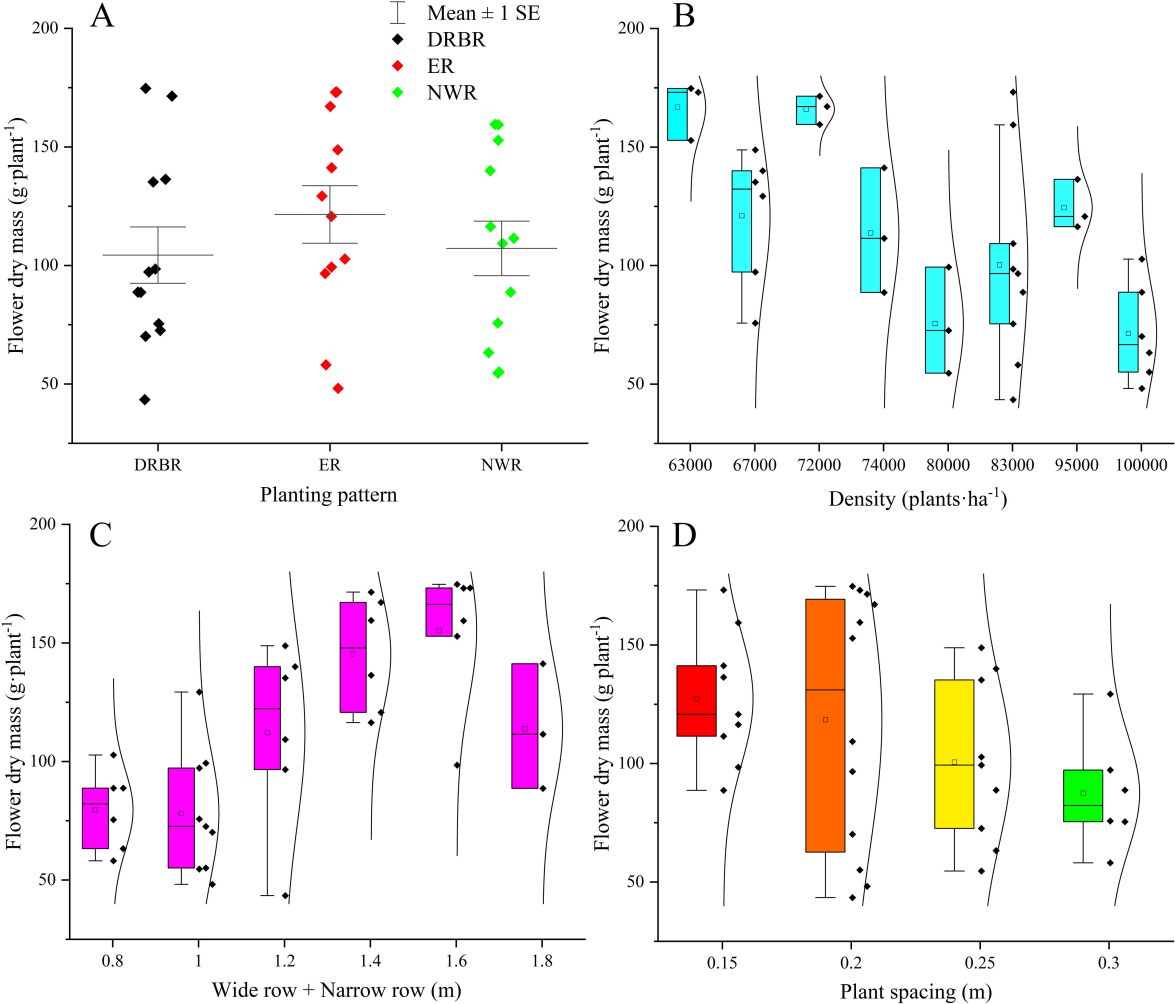
The variations in daylily yield in response to changes in three planting patterns **(A)**, increased planting density **(B)**, the introduction of wide row+narrow row (W+N row) **(C)**, and adjustments in plant spacing **(D)** are examined.

Based on the simulated output of the model, a representative indicator reflecting the merits of daylily planting configurations is the fresh weight of daylily buds. As depicted in **Figure 5A**, within the three planting patterns, the yield per individual daylily plant in the DRBR planting pattern exhibits the greatest fluctuation with changes in plant spacing, with the highest individual plant yield occurring in the DRBR planting pattern. The ER planting pattern demonstrates the highest mean yield per individual daylily plant (121.5 g·plant^−1^), while the data dispersion of the NWR planting pattern is the lowest among the three planting patterns. **Figure 5B** illustrates the variation in individual daylily plant yield with increasing planting density. It is observed that when the planting density remains at 72,000 plants·ha^−1^, the individual daylily plant yield consistently maintains higher values (with an average of 166.0 g·plant^−1^) compared to other planting densities. **Figure 5C** depicts the variation in individual daylily plant yield with increasing W+N row values. It is evident from the graph that as the row spacing increases, the individual daylily plant yield shows an upward trend, with the scenario of W+N row = 1.6 m performing the best. **Figure 5D** illustrates the variation in individual daylily plant yield with increasing plant spacing. It is observed that the highest mean peak occurs at a plant spacing of 0.2 m (with an average of 118.4 g·plant^−1^), although the yield data for the 0.2-m plant spacing scenario exhibit considerable fluctuations, followed by the 0.15-m spacing scenario.

**Figure 6:**
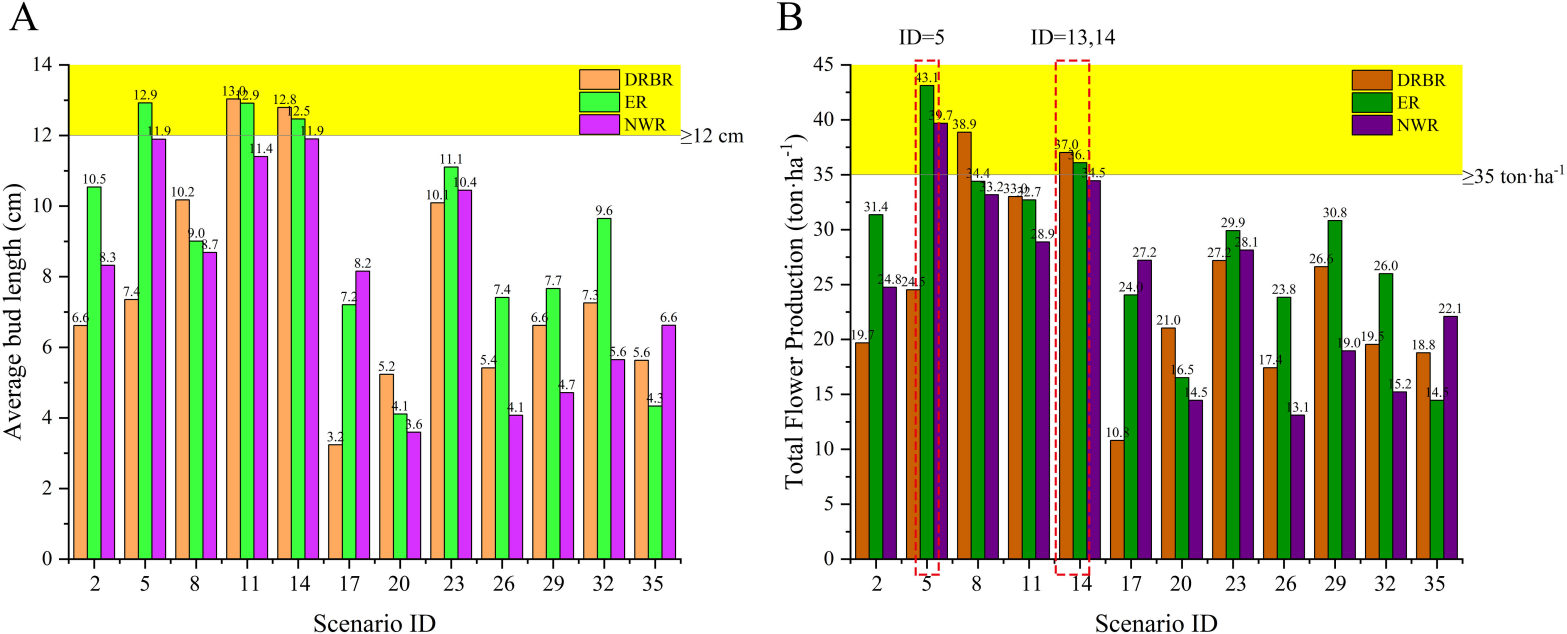
The final average length of daylily buds under various simulated scenario conditions **(A)**. The total yield of daylily buds under different simulated scenario conditions **(B)**.

In practical cultivation, besides the individual bud weight of daylilies, the average length of daylily buds is another crucial indicator affecting daylily sales volume. Generally, daylilies with an average bud length of 12 cm are considered to be of high quality. **Figure 6A** illustrates the average bud length of daylilies under all simulated scenarios. It can be observed from the figure that the average lengths of daylily buds in scenarios with simulation IDs 5, 10, 11, 13, and 14 exceed 12 cm, indicating good quality. **Figure 6B** presents the final total yield of daylilies under all simulated scenarios. The simulation scenarios with yields per hectare greater than or equal to 35 tons are IDs 5, 6, 7, 13, and 14, totaling five scenarios. Combining the results of both simulations, scenarios 5, 13, and 14 meet the requirements for both daylily bud length and total yield. Among them, scenario 13 is the most commonly used planting scenario in production, while scenario 5 is identified as the optimal scenario based on the simulation results.

**Figure 7:**
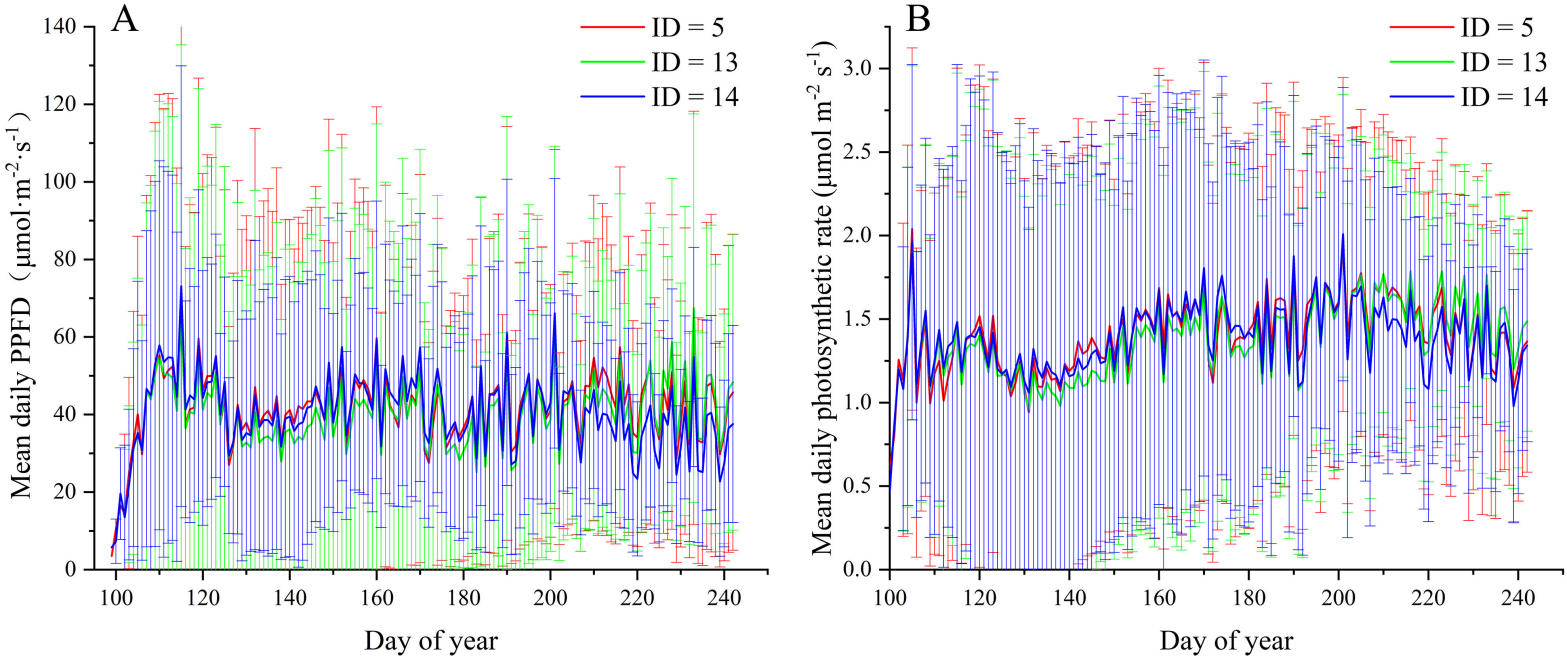
Comparison of daily average solar radiation **(A)** and photosynthetic rate **(B)** in three scenarios (ID = 5, 13, 14).

The three optimal scenarios were selected for comparison. **Figure 7** illustrates the comparison of daily leaf photosynthetic radiation and photosynthetic rate among the three scenarios. As depicted in **Figure 7A**, scenarios 5 and 14 exhibit superior performance to scenario 13 during the transition from the seedling growth stage to the flower scape emergence stage (days 130–150), with average values of light interception ID5 = 39.41 μmol·m^−2^·s^−1^, ID13 = 34.53 μmol·m^−2^·s^−1^, and ID14 = 38.58 μmol·m^−2^·s^−1^. Likewise, **Figure 7B** shows a similar trend, with particularly notable photosynthetic performance during days 140 to 150. During the flowering stage, approximately days 210 to 220, the order of performance is ID5 > ID13 > ID14 (ID5 = 46.18 μmol·m^−2^·s^−1^, ID13 = 41.98 μmol·m^−2^·s^−1^, and ID14 = 36.38 μmol·m^−2^·s^−1^), as observed in **Figure 7B**, with respective rates of ID5 = 1.58 μmol·m^−2^·s^−1^, ID13 = 1.56 μmol·m^−2^·s^−1^, and ID14 = 1.41 μmol·m^−2^·s^−1^. These results indicate that ID5 maintains relatively higher levels of leaf light interception and photosynthetic rate during the two critical periods of yield formation.

**Figure 8:**
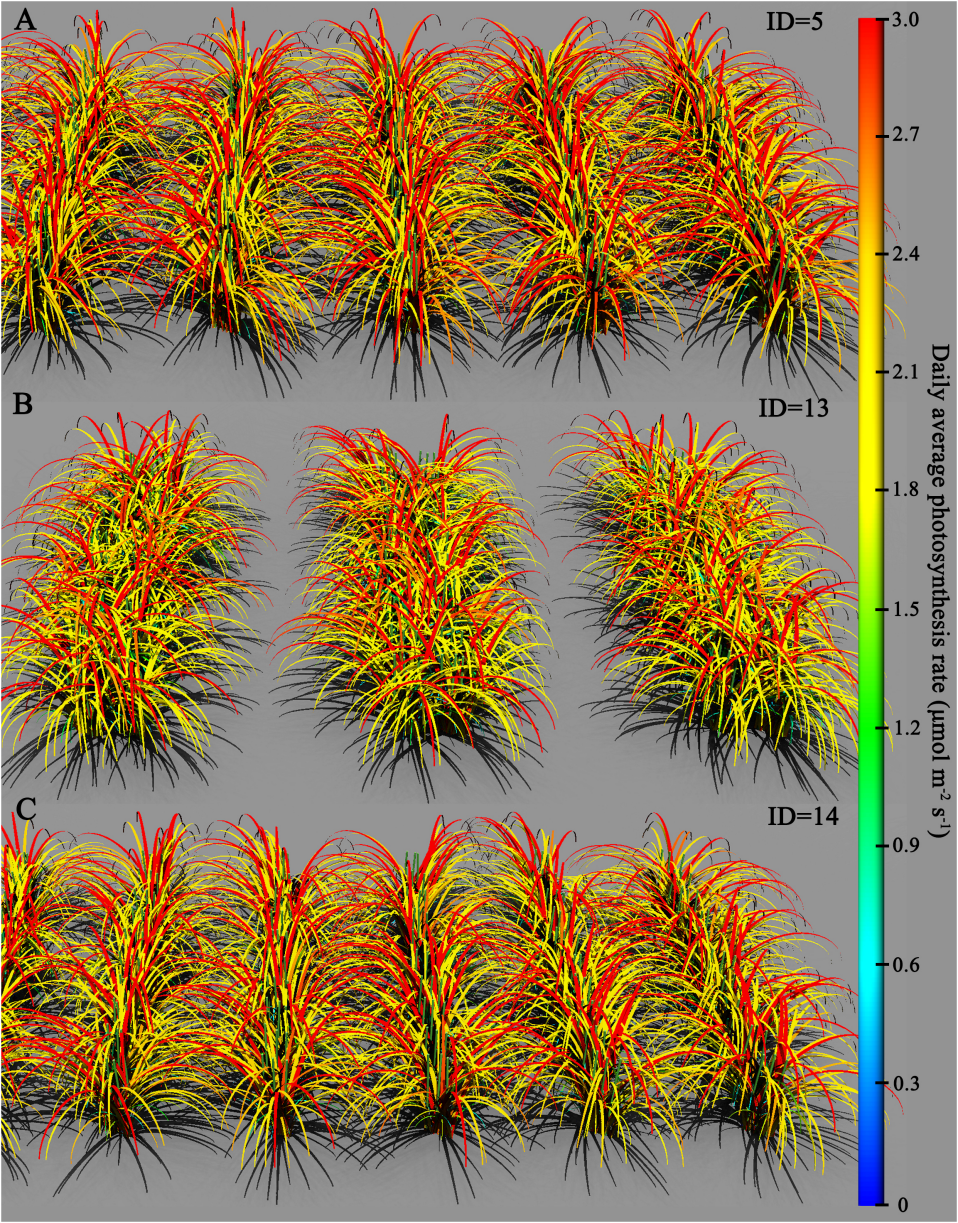
Comparison of daily average photosynthetic rates for different simulated scenarios on the 142nd day: **(A)** ID = 5, **(B)** ID = 13, and **(C)** ID = 14.

**Figure 8** presents the comparison of daily photosynthetic rates among the three scenarios on day 142. From the graph, it is evident that under scenario ID5, the number of leaves with a daily photosynthesis rate approaching 3 μmol·m^−2^·s^−1^ is significantly higher compared to scenarios ID13 and ID14. Moreover, peak photosynthetic rates mainly occur in the upper part of the canopy. Scenario ID13, possibly due to its configuration of large ridges with double rows, exhibits more pronounced shading among plants on the ridges compared to ID5. Consequently, the photosynthetic rate in the upper part of the canopy is not notably high. Both ID14 and ID5 are treated with equal row spacing, with ID14 having a smaller interrow spacing of 0.1 m and a larger plant spacing of 0.05 m compared to ID5. As shown in **Figure 8**, the reduction in interrow spacing in ID14 leads to a greater negative impact on photosynthetic rates compared to the increase in plant spacing by 0.05 m. In the case of ID13, the photosynthetic rates in the lower part of the canopy are relatively low, generally below or equal to 1.2 μmol·m^−2^·s^−1^.

### 3.3 Partial least squares path modeling analysis

To enhance the generalizability of the findings, the PLS-PM method was employed to analyze the interrelationships and causal links between the vegetation configuration characteristics (including planting pattern, row distance, plant spacing, and density) and the physiological features of canopy leaves and buds (including leaf radiation, leaf temperature, leaf photosynthesis, bud length, and dry mass) for all simulated scenarios. The model results indicate that Cronbach’s alpha values are all greater than 0.7, composite reliability values are all greater than 0.7, and the average variance extracted (AVE) is greater than 0.5, confirming the reliability of the model results (**Table 3**).

**Table 3:** Construct reliability and validity.

	Cronbach’s alpha	Composite reliability (rho_a)	Composite reliability (rho_c)	Average variance extracted (AVE)
Bud	1.000	1.000	1.000	1.000
Leaf radiation	0.934	0.958	0.944	0.580
Leaf temperature	0.728	0.706	0.762	0.509
Row distance	0.780	0.720	0.797	0.587
Leaf photosynthesis	0.936	0.952	0.946	0.584

**Figure 9:**
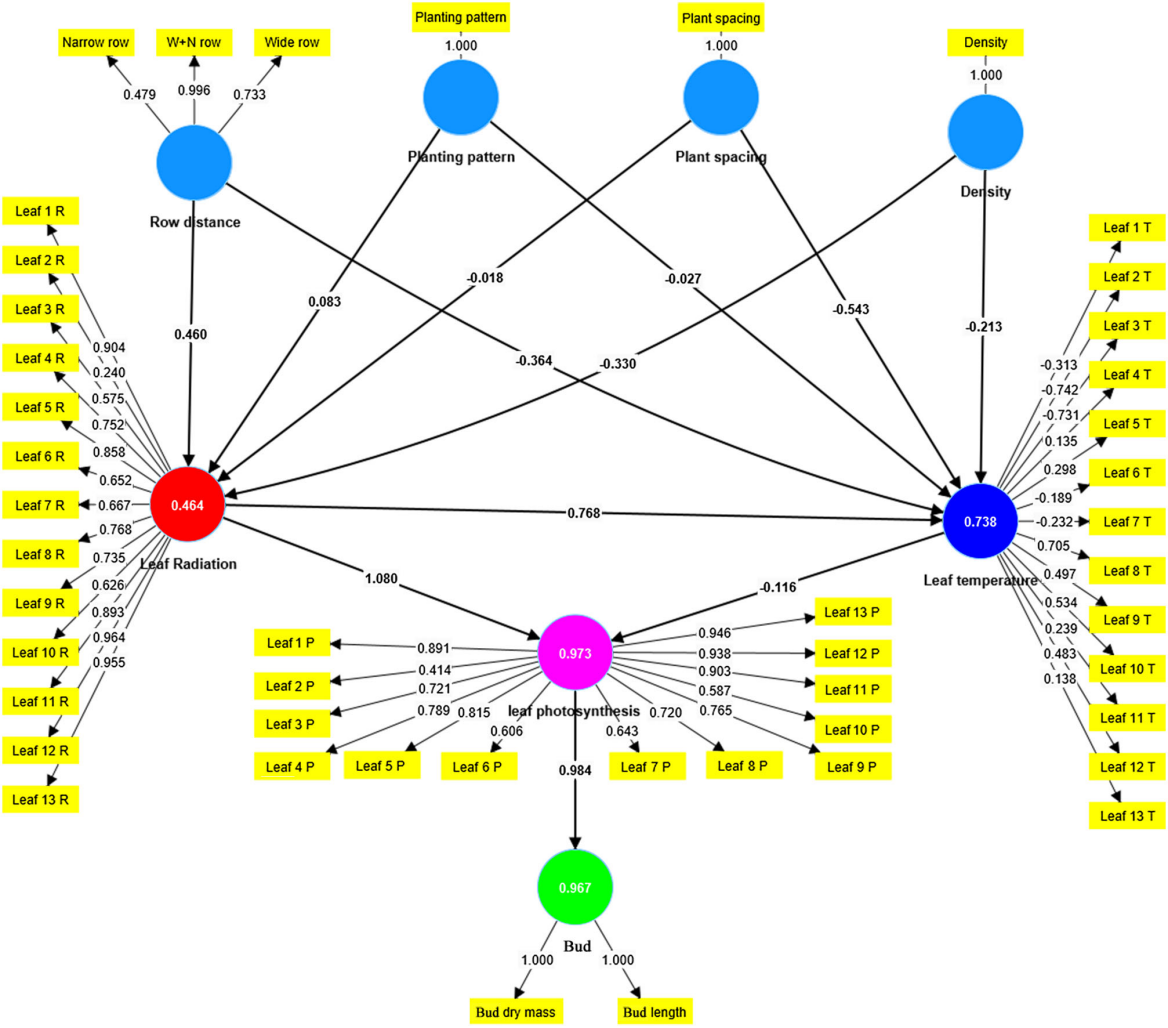
The physiological simulation data and morphological simulation data for all simulated scenarios were statistically analyzed using the partial least squares path modeling (PLS-PM) method implemented in SMARTPLS 4.0 software. In the graphical representation, yellow squares represent all simulated data, blue circles represent latent variables for different vegetation configurations, while other colored circles represent latent variables for leaf light, temperature, photosynthesis, and bud yield.

The analysis reveals that increasing row distance has a relatively pronounced positive effect on canopy light interception (0.460, **Figure 9**), while it has a negative impact on canopy leaf temperature (−0.364). Moreover, it has a positive effect on canopy leaf photosynthesis (0.496) and bud growth (0.488) (**Table 4**). Changing the planting pattern (from DRBR to ER to NWR) has a positive effect on canopy light interception (0.083) and forms a weakly positive effect on bud yield (0.088). Increasing plant spacing in all simulated scenarios leads to a reduction in canopy leaf light interception and leaf temperature (−0.018), with insignificant effects on bud yield (−0.019). Increasing plant density can have a noticeable negative impact on canopy leaf light interception, leaf temperature, and leaf photosynthesis (−0.330, −0.213, and −0.357, respectively), and it can result in a decrease in individual bud yield (−0.351).

**Table 4:** Specific indirect effects between the latent variables.

Specific indirect effects	Specific indirect effects
Density -> leaf temperature -> leaf photosynthesis	0.025
Planting pattern -> leaf radiation -> leaf temperature -> leaf photosynthesis -> bud	−0.007
Leaf radiation -> leaf temperature -> leaf photosynthesis	−0.089
Plant spacing -> leaf temperature -> leaf photosynthesis	0.063
Row distance -> leaf radiation -> leaf photosynthesis -> bud	0.488
Planting pattern -> leaf temperature -> leaf photosynthesis	0.003
Row distance -> leaf temperature -> leaf photosynthesis -> bud	0.041
Row distance -> leaf temperature -> leaf photosynthesis	0.042
Row distance -> leaf radiation -> leaf temperature -> leaf photosynthesis -> bud	−0.040
Density -> leaf radiation -> leaf temperature -> leaf photosynthesis -> bud	0.029
Density -> leaf temperature -> leaf photosynthesis -> bud	0.024
Plant spacing -> leaf radiation -> leaf temperature -> leaf photosynthesis -> bud	0.002
Planting pattern -> leaf radiation -> leaf temperature -> leaf photosynthesis	−0.007
Density -> leaf radiation -> leaf photosynthesis -> bud	−0.351
Leaf radiation -> leaf temperature -> leaf photosynthesis -> bud	−0.087
Plant spacing -> leaf temperature -> leaf photosynthesis -> bud	0.062
Plant spacing -> leaf radiation -> leaf photosynthesis -> bud	−0.019
Density -> leaf radiation -> leaf temperature	−0.254
Density -> leaf radiation -> leaf photosynthesis	−0.357
Plant spacing -> leaf radiation -> leaf temperature	−0.014
Planting pattern -> leaf radiation -> leaf temperature	0.064
Row distance -> leaf radiation -> leaf temperature	0.353
Plant spacing -> leaf radiation -> leaf photosynthesis	−0.020
Planting pattern -> leaf radiation -> leaf photosynthesis -> bud	0.088
Density -> leaf radiation -> leaf temperature -> leaf photosynthesis	0.029
Planting pattern -> leaf radiation -> leaf photosynthesis	0.090
Planting pattern -> leaf temperature -> leaf photosynthesis -> bud	0.003
Row distance -> leaf radiation -> leaf photosynthesis	0.496
Plant spacing -> leaf radiation -> leaf temperature -> leaf photosynthesis	0.002
Row distance -> leaf radiation -> leaf temperature -> leaf photosynthesis	−0.041
Leaf radiation -> leaf photosynthesis -> bud	1.063
Leaf temperature -> leaf photosynthesis -> bud	−0.114

## 4 Discussion

### 4.1 Daylily growth model based on source–sink relationships

In the presented growth model, external climatic variations directly influence the daily growth status of the virtual daylily plants. Altering input parameters such as cloud cover, temperature, relative humidity, and CO_2_ concentration, as well as planting configurations and density, directly impacts the growth status of virtual leaves and flower scapes and even influences the yield of buds during flowering and harvesting periods (i.e., the average length of bud organs). In contrast to other studies on three-dimensional plant population morphology (e.g., Chang et al., 2023), this research not only achieves three-dimensional visualization of daylily organs, individuals, and populations but also establishes light and physiological models and source–sink relationship models tailored for species in the *Hemerocallis* family. This enables precise simulation of daylily yield, rendering the study more comprehensive and detailed than previous research.

Simulations of photosynthetic characteristics in the leaves demonstrate higher rates in early spring due to the gradual emergence and growth of new leaves, with unobstructed sunlight between smaller seedlings. However, considering actual climatic conditions and various planting configurations, actual net photosynthetic rates tend to be lower than ideal conditions, with simulated net assimilation rates ranging mostly between 0.5 and 2.5 μmol CO_2_·m^−2^·s^−1^, aligning closely with the findings of Geng et al. (2023).

While the presented model offers valuable insights into the growth dynamics and yield optimization of daylily plants, it is crucial to recognize its limitations and underlying assumptions. The model’s validation was conducted using field data from a specific region (Datong, Shanxi Province, China), potentially restricting its generalizability to other areas with differing climatic conditions (e.g., different soil types and fertility, water availability, and irrigation practices). To enhance the model’s robustness and applicability, future studies should aim to validate it across a diverse range of geographic locations. Additionally, our model relies on several assumptions concerning physiological processes and growth parameters. For instance, the SLA and growth respiration rates are presumed constant, which may not be accurate under varying environmental conditions. Future research should concentrate on refining these parameters based on empirical data. Furthermore, the simulation results are subject to uncertainties due to the stochastic nature of climatic inputs and the simplifications inherent in the model. For example, the sun and sky radiation module assumes uniform cloud cover, which may not accurately represent actual weather patterns.

### 4.2 Relations between canopy configuration and bud production

In practical agricultural production, the limited land available to farmers necessitates a scientific approach to achieving high yields and quality in daylily cultivation. Balancing density and yield optimally becomes a pressing concern. Thus, we established the commonly used planting density of 72,000 plants·ha^−1^ in Datong, a major Chinese daylily production area, as the baseline, ranging from 63,000 to 100,000 plants·ha^−1^. Various planting patterns and row spacings were simulated to analyze the effects of different densities. Our findings indicate that while planting patterns influence daylily bud yield, the impact is relatively modest, echoing similar conclusions found in other studies. For example, various planting methods influence wheat crop yield to a restricted extent, wherein the application of plastic film and wheat straw mulching exhibits the potential for augmenting wheat yield and improving water utilization efficiency in arid locales (El-Hendawy et al., 2022). However, factors such as competition among plants, soil types, and water management could influence daylily growth and yield. These potential confounding variables might differ among planting patterns, affecting the reliability of our results. Future research should control these variables or reduce their impact through experimental design and statistical analysis methods. Notably, alterations in row spacing (W+N row) tend to increase individual plant yield, whereas plant spacing exhibits no discernible pattern. This observation aligns with our PLS-PM analysis (**Figure 9**) and findings in rice cultivation, where wider spacing of single seedlings has been shown to enhance yield under specific water regimes (Mishra and Salokhe, 2010). Regarding planting density, overall individual daylily plant bud yield gradually decreases with increasing density, mirroring findings in corn cultivation, where higher densities may not yield net benefits (Murphy et al., 1996). However, individual plant yield does not directly correlate with total yield. Thus, this study compares and selects the optimal basic planting configuration (ID5: density of 83,000 plants·ha^−1^, row spacing of 0.8 m, equal row, and plant spacing of 0.15 m) by evaluating the average bud length and total yield across all scenarios. Detailed comparisons of daily average radiation and photosynthetic rates throughout the growth cycle reveal that ID5 consistently outperforms other scenarios (ID13, ID14) during key periods of bud yield formation. This underscores why ID5 emerges as the optimal planting pattern. Thus, our result indicates that, compared to the commonly used initial planting density of 72,000 plants·ha^−1^ in practical agriculture (yielding 37 tons·ha^−1^), the ID5 scenario with a density of 83,000 plants·ha^−1^ increases the planting density by 11,000 plants·ha^−1^ and achieves a yield of 43.1 tons·ha^−1^, thus enhancing the yield by 6.1 tons·ha^−1^. This significantly improves daylily productivity while minimizing land use.

## 5 Conclusion

Reasonable planting patterns are crucial guarantees for high and superior yields of daylilies. Three-dimensional dynamic growth simulation of daylily plants comprehensively presents and expresses the entire process from perceiving external environmental stimuli to germination, growth, and eventual formation of plant morphology and yield. This study focuses on daylily in northern China, utilizing measured data on outdoor climatic conditions, plant growth morphology, and photosynthetic physiology to analyze the quantitative relationships within the daylily source–sink system. Employing the β growth function, growth changes of daylily leaves, scapes, and buds are simulated to construct a dynamic functional–structural growth simulation model of *Hemerocallis* plants. This model facilitates the simulation of daylily bud yields under 36 planting configurations, encompassing three planting patterns, six row spacing patterns, four plant spacings, and 10 initial planting densities. An optimal planting scheme has been identified, the results indicate that scenario ID5, with a density of 83,000 plants·ha^−1^, a row spacing of 0.8 m, and equidistant planting, with a plant spacing of 0.15 m, yields the optimal solution. Furthermore, it is observed that an increase in W+N row spacing to a certain extent accompanies an increase in yield. While planting patterns do influence daylily yield to a certain extent, the extent of this influence is limited, and there is no apparent regularity in the effect of plant spacing on individual plant yield. The simulation results of this study can similarly be applied to other *Hemerocallis* crops.

## Data Availability

The raw data supporting the conclusions of this article will be made available by the authors, without undue reservation.
