# Supplementary material for: Optimizing daylily (Hemerocallis citrina Baroni) cultivation: integrating physiological modeling and planting patterns for enhanced yield and resource efficiency
Source: Front Plant Sci. 2024 Sep 13;15:1442485. doi: 10.3389/fpls.2024.1442485 (PMC11443226; doi:10.3389/fpls.2024.1442485)
Supplement: Supplementary file 1 [file DataSheet1.docx]

Supplementary Material

# Supplementary Figures and Tables

**
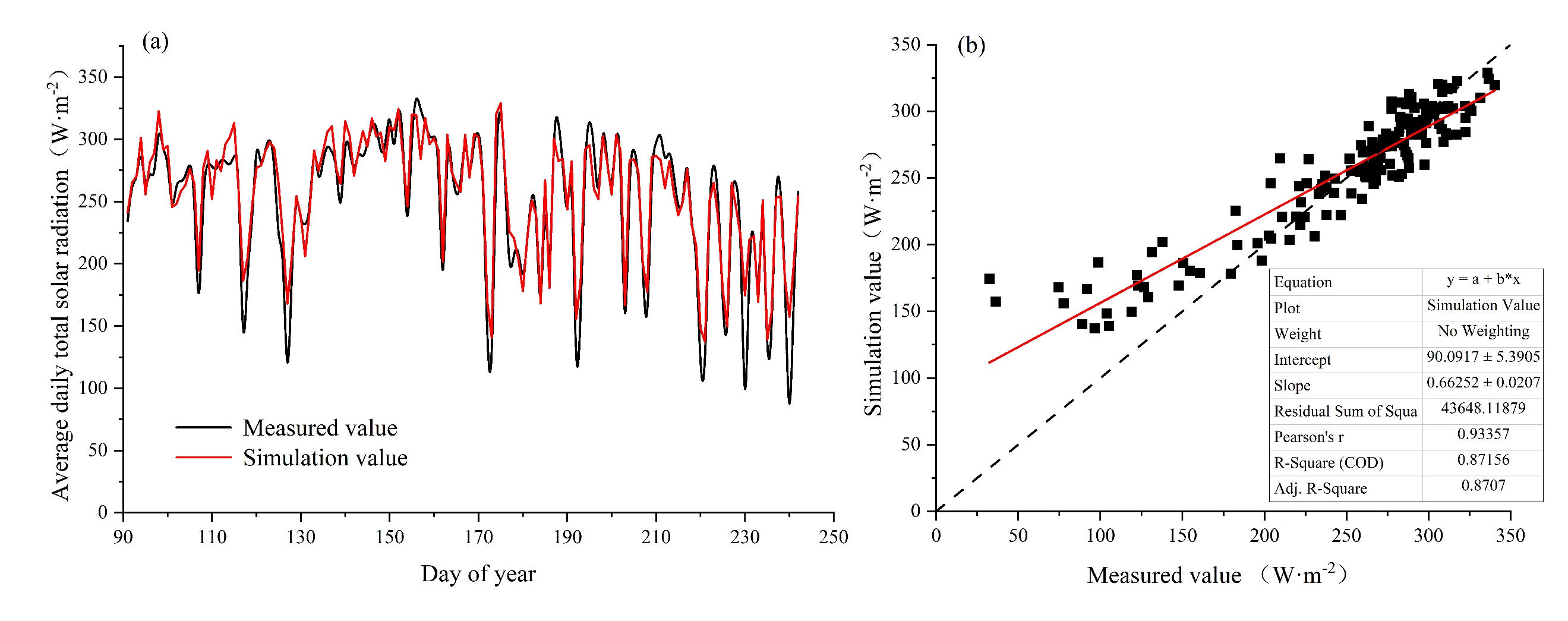
**

**Supplementary Figure.S1** The simulated values of outdoor ground-level solar radiation and the measured values (a), and their evaluation metrics (b).

**Supplementary Table.S1** Equations of the K&L module including photosynthesis (FvCB modified) (Farquhar et al., 1980; Kim and Lieth, 2003), stomatal conductance (BWB modified) (Ball et al., 1987) and energy balance model.

| Model | Equation | Description |
| --- | --- | --- |
| Leaf energy balance model of daylily | $T_{L}=T_{a}+\frac{R_{abs}-\varepsilon\sigma T_{a}^{4}-\lambda g_{v}D/P_{a}}{c_{p}\left( g_{h}+g_{r} \right)+\lambda[\frac{de_{s}(T_{a})/dT}{P_{a}}]g_{v}}$ | Energy balance for leaf temperature $T_{L}$ |
|  | $g_{r}=\frac{4\varepsilon\sigma T_{L}^{3}}{C_{p}}$ | Leaf emitted thermal radiation |
|  | $g_{h}=g_{b}\times0.135/0.147$ | Leaf boundary layer conductance for heat transfer |
|  | $g_{v}=\frac{0.5\times g_{s}g_{b}}{g_{s}+g_{b}}$ | Leaf boundary layer conductance |
|  | $e_{s}\left( T \right)=0.611 \exp(\frac{17.502T}{240.97+T})$ | Leaf saturation vapour pressure at *T* |
|  | $e=2g_{V}\left[ \frac{e_{s}(T_{L})-e_{a}}{P_{a}} \right]$ | Leaf transpiration rate |
| Photosynthesis model (FvCB model) | $A=V_{c}-0.5V_{0}-R_{d}=\min\left\{ A_{c}，A_{j}，A_{p} \right\}-R_{d}$ | Net photosynthetic rate |
|  | $A_{c}=V_{cmax}\frac{C_{c}-\Gamma_{*}}{C_{c}+K_{c}(1+O/K_{o})}$ | Rubisco-limited photosynthetic rate |
|  | $A_{j}=\frac{J(C_{i}-\Gamma_{*})}{4(C_{i}+2\Gamma_{*})}$ | RuBP regeneration limited photosynthetic rate |
|  | $\theta J^{2}-\left( I_{2}+J_{\max} \right)J+I_{2}J_{\max}=0$ | Light dependence of the rate of electron transport |
|  | $I_{2}=I(1-f)(1-\delta)/2$ | PAR effectively absorbed by Photosystem II |
|  | $A_{p}=3P_{u}$ | TPU limited photosynthetic rate |
|  | $K_{T}=k_{25}\exp\left[ \frac{E_{a}(T_{L}-25)}{298R\left( T_{L}+273 \right)} \right]$ | Arrhenius function; temperature dependence of $K_{c}$, $K_{o}$, $R_{d}$, $V_{cmax}$ |
|  | $J_{max}=J_{m25}\exp\left[ \frac{\left( T_{p}-25 \right)E_{a}}{R\left( T_{p}+273 \right)298} \right]\left\{ \frac{1+\exp\left( \frac{S298-H}{R298} \right)}{1+\exp\left[ \frac{S\left( T_{p}+273 \right)-H}{R(T_{p}+273)} \right]} \right\}$ | Temperature dependence of $J_{max}$ |
|  | $\Gamma^{*}=36.9+1.88\left( T_{p}-25 \right)+0.036{(T_{p}-25)}^{2}$ | Temperature dependence of $\Gamma^{*}$ |
|  | $f\left( \xi\right)=d_{0}\left[ 1-\exp\left( -d_{1}\xi\right) \right]\exp(-d_{2}\xi)$ | Leaf age dependence of $J_{max}$, $V_{cmax}$ and $P_{u}$ |
|  | $C_{i}=C_{a}-A(\frac{1}{g_{b}}+\frac{1}{g_{s}})P_{a}$ | Intercellular CO_2_ concentration |
|  | $C_{c}=C_{i}-\frac{A}{g_{m}}P_{a}$ | Chloroplastic CO_2_ concentration |
|  | $g_{m}=\frac{\exp(c-\frac{\Delta H_{a}}{RT_{k}})}{1+\exp(\frac{\Delta S\times T_{k}-\Delta H_{d}}{RT})}$ | Mesophyll conductance to CO_2_ |
| Stomatal conductance model (BWB) | $g_{s}=b+mA\frac{h_{s}}{(C_{s}/P_{a})}$ | Stomatal conductance |
|  | $\Gamma=\frac{R_{d}\left[ K_{c}(1+O/K_{o}) \right]+V_{c\max}\Gamma_{*}}{V_{c\max}-R_{d}}$ | CO_2_ compensation point in the presence of R_d_ |
|  | $C_{s}=C_{a}-\frac{A}{g_{b}}P_{a}$ | Estimation of CO_2_ partial pressure at the leaf surface |
|  | $g_{b}=0.147\sqrt{u/d}$ (*Gr/Re^2^*>*1*)  $g_{b}=0.055\cdot{(\frac{T_{p}-T_{a}}{d})}^{\frac{1}{4}}$ (*Gr/Re^2^*<*1*)  $d = 0.72w$ | Boundary layer conductance for water vapor |
|  | $a_{h}h_{s}^{2}+b_{h}h_{s}+c_{h}=0$ 其中$\left\{ \begin{aligned} a_{h}=(g_{1}A)/C_{s} \\ b_{h}=g_{0}+g_{b}-(g_{1}A/C_{s}) \\ c_{h}=\left( -RHg_{b} \right)-g_{0} \end{aligned} \right.$ | Quadratic equation to obtain $h_{s}$by combining $g_{s}$with diffusion equation |

**Supplementary Table.S2** Nomenclature

| **Classification** | **Symbol** | **Meaning and Unit** |
| --- | --- | --- |
| Photosynthesis | $\Gamma$ | CO_2_ compensation point in the presence of R_d_ [μbar] |
|  | $\Gamma^{*}$ | CO_2_ compensation point in the absence of R_d_ [μbar] |
|  | $\xi$ | Leaf age counted as days after unfolding [day] |
|  | $\theta$ | Curvature of response of electron transport to PAR [0.7，-] |
|  | $\delta$ | Leaf reflectance plus transmittance [0.15，-] |
|  | $A$ | Net photosynthetic rate [μmol m^-2^ s^-1^] |
|  | $A_{c}$ | Rubisco-limited CO_2_ assimilation rate [μmol m^-2^ s^-1^] |
|  | $A_{j}$ | Electron transport-limited CO_2_ assimilation rate [μmol m^-2^ s^-1^] |
|  | $A_{\max}$ | Light-saturated CO_2_ assimilation rate at ambient [CO_2_] [μmol m^-2^ s^-1^] |
|  | $A_{p}$ | Triose phosphate utilization-limited CO_2_ assimilation rate [μmol m^-2^ s^-1^] |
|  | $C_{a}$ | Ambient CO_2_ partial pressure |
|  | $C_{i}$ | Intercellular CO_2_ partial pressure [μbar] |
|  | $d_{0}$ | Scaling factor of leaf age effect [1.296，-] |
|  | $d_{1}$ | Empirical coefficient to determine growth of leaf age effect [0.1468，-] |
|  | $d_{2}$ | Empirical coefficient to determine downward slope of leaf age effect [0.0103，-] |
|  | $E_{a}$ | Activation energy [kJ mol^-1^] |
|  | $f$ | Spectral correction factor [0.15，-] |
|  | $H$ | Curvature parameter of the temperature dependence $J_{\max}$ [219.4 kJ mol^-1^] |
|  | $I$ | Incident PAR [μmol photons m^-2^ s^-1^] |
|  | $J$ | Electron transport rate [μmol electrons m^-2^ s^-1^] |
|  | $J_{m25}$ | Potential rate of electron transport at 25°C [36.8 μmol m^-2^ s^-1^] |
|  | $K_{c25}$ | Michaelis-Menten constant of Rubisco for CO_2_ [267 μbar] |
|  | $K_{o25}$ | Michaelis-Menten constant of Rubisco for O_2_ [164 mbar] |
|  | *O* | Oxygen partial pressure [205 mbar] |
|  | $P_{u25}$ | Rate of triose phosphate utilization at 25°C [10.35 μmol m^-2^ s^-1^] |
|  | *R* | Universal gas constant [8.314 J mol^-1^ K^-1^] |
|  | $R_{d25}$ | Mitochondrial respiration in the light at 25°C [0.0089*V_cmax25_*] |
|  | *S* | Electron transport temperature response parameter [704.2 J mol^-1^ K^-1^] |
|  | $V_{c}$ | Carboxylation rate [μmol m^-2^ s^-1^] |
|  | $V_{cm25}$ | Photosynthetic Rubisco capacity at 25°C [92.07 μmol m^-2^ s^-1^] |
|  | $V_{o}$ | Oxygenation rate [μmol m^-2^ s^-1^] |
| Variables with temperature dependence (E for activation energy of each process) | E | Maximum rate of electron transport [μmol m^-2^ s^-1^] |
|  | $J_{\max}$ | Michaelis-Menten constant of Rubisco for CO_2_ [μmol m^-2^ s^-1^] E_Kc_ 80990 |
|  | $K_{c}$ | Michaelis-Menten constant of Rubisco for O_2_ [μmol m^-2^ s^-1^] E_Ko_ 36000 |
|  | $K_{o}$ | Triose phosphate utilization rate [μmol m^-2^ s^-1^] E_Pu_ 47140.1 |
|  | $P_{u}$ | Mitochondrial respiration in the light [μmol m^-2^ s^-1^] E_Rd_ 46390 |
|  | $R_{d}$ | Maximum rate of Rubisco carboxylation [μmol m^-2^ s^-1^] E_Vcmax_ 65330 |
|  | $V_{c\max}$ | Maximum rate of electron transport [μmol m^-2^ s^-1^] |
| Stomatal conductance model | *b* | Minimum stomatal conductance to water vapour at the light compensation point in the BWB model [0.0960 mol m^-2^ s^-1^] |
|  | $C_{a}$ | Ambient CO_2_ partial pressure [μbar] |
|  | $C_{s}$ | CO_2_ partial pressure at the leaf surface [μbar] |
|  | $g_{b}$ | Boundary layer conductance to water vapour [mol m^-2^ s^-1^] |
|  | $g_{s}$ | Stomatal conductance to water vapour [mol m^-2^ s^-1^] |
|  | $g_{h}$ | Leaf boundary layer conductance for heat transfer [mol m^-2^ s^-1^] |
|  | $g_{v}$ | Leaf boundary layer conductance [-] |
|  | $h_{s}$ | Relative humidity at the leaf surface [-] |
|  | *m* | Empirical coefficient for the sensitivity of g_s_ to A，C_s_ and h_s_ in the BWB model [10.055，-] |
| Energy balance model | $\varepsilon$ | Leaf thermal emissivity [0.97] |
|  | $\sigma$ | Stefan-Boltzmann constant per surface area [$5.67\times{10}^{-8} W m^{-2}K^{-4}$] |
|  | $\lambda$ | Latent heat of vaporization at 25 ℃ [44.0 kJ mol^-1^] |
|  | $c_{p}$ | Specific heat of air [29.3 J mol^-1^ C^-1^] |
|  | *D* | Vapour pressure deficit of the ambient air [kPa] |
|  | $D_{s}$ | Vapour pressure deficit at the leaf surface [kPa] |
|  | *E* | Transpiration rate per leaf area [mol m^-2^ s^-1^] |
|  | $e_{a}$ | Vapour pressure in the ambient air [kPa] |
|  | $e_{s}$ | Vapour pressure at the leaf surface [kPa] |
|  | $g_{h}$ | Heat conductance for boundary layer per surface leaf area [mol m^-2^ s^-1^] |
|  | $g_{r}$ | Radiative conductance per surface leaf area [mol m^-2^ s^-1^] |
|  | $g_{v}$ | Total water vapour conductance per surface leaf area [mol m^-2^ s^-1^] |
|  | $P_{a}$ | Atmospheric pressure [kPa] |
|  | $R_{abs}$ | Absorbed long-wave and short-wave radiation per surface leaf area [W m^-2^] |
|  | $T_{a}$ | Air temperature ℃ |
